# Controlling nutritional status score in the prediction of cardiovascular disease prevalence, all-cause and cardiovascular mortality in chronic obstructive pulmonary disease population: NHANES 1999–2018

**DOI:** 10.1186/s12890-024-03175-7

**Published:** 2024-07-24

**Authors:** Suying Mai, Yayun Nan, Linlin Peng, Yuanbo Wu, Qiong Chen

**Affiliations:** 1 Department of Geriatrics, Xiangya Hospital, Central South University, Changsha, China; 2 National Clinical Research Center for Geriatric Disorders, Xiangya Hospital, Central South University, Changsha, China; 3 Department of Ningxia Geriatrics Medical Center, Ningxia People’s Hospital, Yinchuan, China

**Keywords:** Chronic obstructive pulmonary disease, Controlling nutritional status (CONUT) score, Cardiovascular disease, Malnutrition, Mortality

## Abstract

**Background:**

Malnutrition is prevalent in chronic obstructive pulmonary disease (COPD) and associated with adverse outcomes, while COPD is intricately linked to cardiovascular disease (CVD), sharing common risk factors. The controlling nutritional status (CONUT) score, a promising tool for assessing malnutrition, warrants investigation into its predictive ability for cardiovascular disease prevalence and mortality in COPD patients.

**Methods:**

Based on the National Health and Nutrition Examination Survey (NHANES), this study analyzed 1501 adult COPD patients from 1999 to 2018. The endpoints were CVD prevalence, mortality related to CVD, and overall mortality. We evaluated the correlation of the CONUT score with each outcome using logistic regression and Cox regression models. The prognostic evaluation of patients was conducted using Kaplan-Meier curves in accordance with the CONUT score. We formed the receiver operating characteristic (ROC) curves for evaluating the CONUT score's discriminative capability.

**Results:**

The prevalence of malnutrition was 21.31% in COPD populations. Logistic analyses suggested a distinct connection between the CONUT score and CVD prevalence (OR:1.86, 95%CI:1.28-2.70) in individuals with COPD. The CONUT score demonstrated a significant correlation with a heightened risk of CVD mortality (HR: 1.86, 95%CI: 1.27-2.74) and overall mortality (HR: 1.50, 95%CI: 1.18-1.91). The prognostic outcomes might be effectively discriminated by the CONUT score, as seen by the Kaplan-Meier curves.

**Conclusions:**

In summary, the CONUT score provides an uncomplicated and readily attainable marker for forecasting CVD prevalence, total mortality, and mortality from CVD among COPD patients.

**Supplementary Information:**

The online version contains supplementary material available at 10.1186/s12890-024-03175-7.

## Background

According to GOLD 2023, chronic obstructive pulmonary disease (COPD) involves impairments in the respiratory system that cause constant and often worsening air flow restriction on account of airway and/or alveolar abnormalities [1]. COPD is recognized as the third most significant contributor to worldwide mortality, underscoring its profound impact on public health [2]. Emerging evidence suggests that malnutrition is a prevalent and influential comorbidity in COPD, with reported prevalence rates ranging from 20 to 60% [3]. Malnutrition in COPD is multifactorial, attributed to factors such as decreased dietary intake due to dyspnea during meals, increased energy expenditure, aging, muscle loss and atrophy, tissue hypoxia, and systemic inflammation [4, 5]. Furthermore, malnutrition has been linked to adverse clinical outcomes in COPD, including worsened lung function, heightened exacerbation frequency, prolonged hospitalizations, and increased mortality rates [3, 4]. It significantly affects muscle energetics, exercise tolerance, and the severity of dyspnea, three aspects crucial to the overall well-being and living quality of COPD patients [5]. Therefore, understanding and addressing malnutrition in COPD patients are imperative for optimizing clinical management and improving outcomes.

In addition to malnutrition, COPD is intricately associated with cardiovascular disease (CVD), which is one of the most common and serious complications. COPD and CVD share common risk factors and pathophysiological mechanisms [1]. Classic CVD risk factors prevalent in COPD, such as smoking, inflammation, and oxidative stress, contribute to the development and progression of cardiovascular complications. Moreover, COPD-related factors, including chronic hypoxemia, pulmonary hypertension, and systemic inflammation, further exacerbate cardiovascular dysfunction and increase the risk of adverse cardiovascular events [6, 7]. Malnutrition-induced alterations in immune function, oxidative stress, and endothelial dysfunction may exacerbate cardiovascular complications in COPD [5]. Given the complex interplay between COPD, malnutrition, and cardiovascular disease, there is growing interest in exploring the potential role of malnutrition as a contributing factor to cardiovascular risk in COPD patients. Therefore, identifying and addressing malnutrition in COPD patients may have implications for reducing cardiovascular risk and improving overall prognosis.

Recently, the controlling nutritional status (CONUT) score has emerged as a promising tool for assessing malnutrition in hospitalized patients, incorporating serum albumin, lymphocyte count, and total cholesterol levels [8]. The CONUT score provides valuable insights into nutritional status, immune function, and lipid metabolism, making it a comprehensive indicator of malnutrition and associated complications [9–11]. While the prognostic value of the CONUT score has been demonstrated in various medical conditions, including cancer, cardiac failure, and kidney disease, its utility in COPD remains underexplored [12–14]. Recent literature suggests that the CONUT score may also have predictive capabilities for exacerbations of COPD [15]. Therefore, this research aims to investigate the predictive ability of the CONUT score for cardiovascular disease prevalence and mortality in COPD patients, shedding light on its potential clinical utility in this population.

## Methods

### Study population

The National Health and Nutrition Examination Survey (NHANES) employs a categorized, multistage probabilistic design to gather data. The information involves conducting interviews, health screenings, and laboratory sample analysis on representative samples [16]. NHANES offers an evaluation of the dietary and health conditions of the people in the United States.

**Fig. 1:**
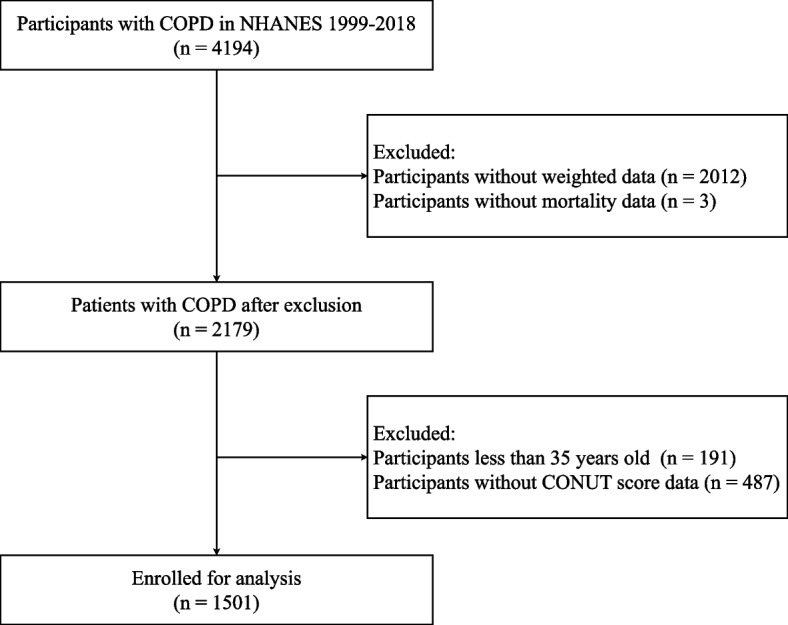
Flowchart of enrolling study cohort. COPD, chronic obstructive pulmonary disease; NHANES, National Health and Nutrition Examination Survey; BMI, body mass index

We included all people ≥ 35 years of age with COPD from NHANES 1999-2018. The subsequent elements were employed to determine candidate exclusion: (1) Participants without weighted data. (2) Participants without CONUT score data. (3) Participants without mortality data. Ultimately, 1501 patients with COPD were incorporated, as illustrated by the flowchart (Fig. 1).

### Definition of CONUT score

The CONUT score serves as an indicator for evaluating the nutrition and immune-related situation by considering lymphocyte composition, total cholesterol, and albumin levels [8]. Normal nutrition level is represented by a count of 0 or 1, mild malnutrition is indicated by 2 to 4, moderate malnutrition is denoted by 5 to 8, and severe malnutrition is indicated by 9 to 12. The enrolled participants were classified into two distinct groups: those who exhibited normal nutrition (CONUT: 0–1) and those who suffered from malnutrition (CONUT ≥ 2).

### Diagnosis of COPD and CVD

The diagnosis of COPD relied on the NHANES questionnaire (medical conditions dataset) data files. COPD was confirmed based on the subsequent inquiries: “Ever told you had COPD by doctors”, “Ever told you had chronic bronchitis by doctors”, and “Ever told you had emphysema by doctors”. Individuals who answered affirmatively to any of the three inquiries were classified as those diagnosed with COPD [17, 18].

To confirm the CVD, the medical conditions questionnaire was administered. The questionnaire included “Ever told had congestive heart failure”, “Ever told you had coronary heart disease”, “Ever told you had angina/angina pectoris”, “Ever told you had heart attack” and “Ever told you had a stroke”. Individuals would be issued a diagnosis of CVD if they replied positively to any one of the five inquiries [19, 20].

### Covariate assessments

A number of probable interfering variables, including age, race, sex, body mass index (BMI), smoking status, education, hypertension, diabetes, asthma and cardiovascular-related diseases, were incorporated into our study. The classification of race encompassed those who identified as non-Hispanic black, Mexican American, non-Hispanic white, or belonging to any other racial category. Age spread was the following: over 60 years and under 60 years. Degrees of education were delineated as college graduate or above, high school graduate or equivalent, 9-11th grade, and less than 9th grade. The situation of cigarette usage might be classified into three distinct categories: those who have never engaged in smoking, individuals who used to smoke but have since quit, and individuals who now smoke. BMI is determined by dividing a person's weight by the square of his or her height. BMI has been classified into several categories, namely normal (18.5–24.9 kg/m^2^), underweight (< 18.5 kg/m^2^), overweight (25.0–29.9 kg/m^2^), and obesity (≥ 30.0 kg/m^2^) [21].

### Outcomes

Our study outcomes were CVD prevalence, all-cause, and cardiovascular mortality. The mortality was confirmed upon the acquisition of records sourced from the National Death Index, and the due date for follow-up was December 31, 2018. Cardiovascular mortality was defined by considering fatalities attributed to cardiac and cerebrovascular diseases, as indicated by the assigned codes: UCOD_LEADING = 001 (‘disease of the heart’) or 005 (‘cerebrovascular disease’).

### Statistical analysis

NHANES developed weighting procedures to address the intricate survey layout, failure to respond issues, and post-stratification modifications necessary for achieving representativeness of the whole population of the United States. Consequently, based on the NHANES analytic recommendations, we applied sample weighting in the bulk of the subsequent analyses [22]. Given that the proportion of missing values was less than 3%, these missing values were interpolated using a weighted median and mode. In order to compare continuous variables denoted by weighted means ± standard error (SE), the t-test was applied. With the aid of the chi-square test, comparative analysis was conducted on categorical variables represented as numbers (percentages). The association between age and the prevalence of malnutrition was examined by the Cochran-Armitage trend test utilizing the *p* for trend. Using logistic regression models, the research calculated odds ratios (ORs) and 95% confidence intervals (CIs) for the correlation between the CONUT score and CVD prevalence. To evaluate the associations between CONUT score and mortality, hazard ratios (HRs) and 95% CIs were calculated with an estimation of Cox proportional risk regression models. In the context of multivariate analysis, the elements that exhibited statistical signification in the univariate analysis were subjected to adjustment. The survival rates of different CONUT score categories were examined through the implementation of the Kaplan–Meier survival analysis. The area under the receiver operating characteristic (ROC) curves (AUCs) were calculated subsequent to the generation of ROC curves. Thus, the CONUT score was assessed for its performance in predicting CVD prevalence, CVD mortality, and total mortality prediction. Subsequently, subgroup analyses were conducted, employing stratification based on aging, gender, ethnicity, smoking status, educational attainment, diabetes, hypertension, asthma and BMI. The total outcomes were statistically evaluated using the R program (version 4.3.2). As the two-sided *p*-value < 0.05, statistical significance was considered to be present.

## Results

### Baseline characteristics

The study encompassed a cohort of 1501 individuals with a median follow-up duration of 117 months who received a diagnosis of COPD. Additional file 1: Figure S1 demonstrated that there is a tendency for the prevalence of malnutrition in individuals to increase with age (p for trend < 0.001). Table 1 displayed the fundamental attributes of the entire cohort, as well as two distinct groups classified according to varying COUNT score levels. The prevalence of malnutrition (CONUT score ≥ 2) was 21.31% in COPD populations after weighting. A substantially different distinction was observed in several health indicators between the malnourished and normal nutrition groups, including age, sex, smoking status, diabetes, CVD, stroke, congestive heart failure, hypertension, coronary heart disease, heart attack, and angina (*p* < 0.05). Regarding race, BMI, asthma, and education, there were no statistical variations.

**Table 1:** Characteristics of patients with COPD

Characteristics	Overall (*n* = 1501)	CONUT 0–1 (*n* = 1144)	CONUT ≥ 2 (*n* = 357)	*p* value
Age				< 0.001
< 60	657 (53.27)	562 (57.23)	95 (38.62)	
≥ 60	844 (46.73)	582 (42.77)	262 (61.38)	
Sex				0.016
Male	632 (37.72)	438 (35.82)	194 (44.73)	
Female	869 (62.28)	706 (64.18)	163 (55.27)	
Race				0.187
Non-Hispanic white	933 (79.44)	713 (80.10)	220 (77.00)	
Non-Hispanic black	243 (8.36)	188 (8.46)	55 (7.98)	
Mexican American	131 (3.02)	108 (3.10)	23 (2.75)	
Other races	194 (9.18)	135 (8.34)	59 (12.27)	
Education				0.304
College graduate or above	653 (47.69)	505 (47.22)	148 (49.45)	
High school graduate or equivalent	380 (28.54)	294 (29.91)	86 (23.50)	
9-11th grade	276 (16.46)	199 (15.84)	77 (18.74)	
Less than 9th grade	192 (7.30)	146 (7.03)	46 (8.30)	
BMI				0.357
Normal	306 (20.72)	234 (20.24)	72 (22.47)	
Underweight	44 (3.51)	28 (3.00)	16 (5.41)	
Overweight	438 (29.72)	338 (30.54)	100 (26.68)	
Obese	713 (46.05)	544 (46.22)	169 (45.43)	
Smoke				< 0.001
Never	441 (28.23)	340 (27.01)	101 (32.75)	
Former	555 (35.03)	388 (33.33)	167 (41.28)	
Current	505 (36.74)	416 (39.66)	89 (25.97)	
Cardiovascular Disease				< 0.001
No	1022 (72.56)	833 (76.54)	189 (57.87)	
Yes	479 (27.44)	311 (23.46)	168 (42.13)	
Congestive heart failure				< 0.001
No	1298 (89.61)	1034 (92.66)	264 (78.35)	
Yes	203 (10.39)	110 (7.34)	93 (21.65)	
Coronary heart disease				< 0.001
No	1304 (88.01)	1019 (90.27)	285 (79.66)	
Yes	197 (11.99)	125 (9.73)	72 (20.34)	
Heart attack				< 0.001
No	1286 (87.92)	1012 (90.72)	274 (77.56)	
Yes	215 (12.08)	132 (9.28)	83 (22.44)	
Angina				0.006
No	1333 (89.08)	1035 (90.67)	298 (83.21)	
Yes	168 (10.92)	109 (9.33)	59 (16.79)	
Stroke				0.014
No	1352 (91.40)	1044 (92.53)	308 (87.23)	
Yes	149 (8.60)	100 (7.47)	49 (12.77)	
Hypertension				0.009
No	594 (45.34)	480 (47.78)	114 (36.31)	
Yes	907 (54.66)	664 (52.22)	243 (63.69)	
Diabetes				< 0.001
No	1165 (82.00)	930 (84.00)	235 (74.64)	
Yes	336 (18.00)	214 (16.00)	122 (25.36)	
Asthma				0.200
No	860 (57.38)	648 (58.39)	212 (53.68)	
Yes	641 (42.62)	496 (41.61)	145 (46.32)	

*Abbreviations*: *COPD* chronic obstructive pulmonary disease, *CONUT* controlling nutritional status score, *BMI* body mass index

### Association analysis outcomes

To explore the relationship between CONUT score and CVD prevalence, logistic regression models were used. The covariates were determined by univariate logistic regression (Additional file 2: Table S1). There was a statistically significant association between those with a CONUT score greater than two and an increased incidence of CVD, according to Table 2. Despite accounting for possible interfering covariates, including age, sex, race, education, BMI, smoke, hypertension and diabetes, the observed connection maintained statistical significance (OR:1.86, 95%CI:1.28-2.70). It was also significant in the continuous variable (OR: 1.35, 95%CI:1.16-1.58).

**Table 2:** Multivariate logistic regression analyses of the CONUT score and CVD prevalence in COPD

	Model 1		Model 2		Model 3	
	OR (95%CI)	*p* value	OR (95%CI)	*p* value	OR (95%CI)	*p* value
CONUT score						
< 2 score	ref		ref		ref	
≥ 2 score	2.00 (1.42-2.81)	< 0.001	1.98 (1.40-2.81)	< 0.001	1.86 (1.28-2.70)	0.001
Continuous (per 1-unit higher)	1.40 (1.22-1.61)	< 0.001	1.40 (1.21-1.61)	< 0.001	1.35 (1.16-1.58)	< 0.001

Model 1: adjusted for age and sex. Model 2: adjusted for age, sex, race and education. Model 3: adjusted for age, sex, race, education, smoke, BMI, hypertension and diabetes

*Abbreviations*: *CONUT* controlling nutritional status score, *CVD* cardiovascular disease, *COPD* chronic obstructive pulmonary disease, *OR* odds ratios, *CI* confidence interval, *BMI* body mass index

Cox regression analyses were performed with the objective of examining the correlation between the CONUT score and mortality by adjusting for univariate analysis of significant variables (Additional file 3: Table S2). Following the adjustment for all covariates, Table 3 showed that the CONUT score ≥ 2 group was significantly related to cardiovascular mortality (HR:1.86, 95%CI:1.27-2.74) and overall mortality (HR: 1.50, 95%CI:1.18-1.91). The continuous variable was of equal significance. The survival curves (Additional file 4: Figure S2) demonstrated that the CONUT score had a well-differentiated risk capability for both overall mortality and CVD mortality (*p* < 0.0001).

**Table 3:** Multivariate COX regression analyses of the CONUT score and overall and cardiovascular mortality in COPD

	Model 1		Model 2		Model 3	
	HR (95%CI)	*p* value	HR (95%CI)	*p* value	HR (95%CI)	*p* value
**All-cause mortality**						
CONUT score						
< 2 score	ref		ref		ref	
≥ 2 score	1.58 (1.24-2.00)	< 0.001	1.59 (1.24-2.05)	< 0.001	1.50 (1.18-1.91)	< 0.001
Continuous (per 1-unit higher)	1.36 (1.21-1.52)	< 0.001	1.36 (1.21-1.53)	< 0.001	1.30 (1.15-1.47)	< 0.001
**Cardiovascular mortality**						
CONUT score						
< 2 score	ref		ref		ref	
≥ 2 score	2.23 (1.53-3.26)	< 0.001	2.28 (1.54-3.36)	< 0.001	1.86 (1.27-2.74)	0.002
Continuous (per 1-unit higher)	1.48 (1.28-1.70)	< 0.001	1.46 (1.27-1.69)	< 0.001	1.32 (1.15-1.52)	< 0.001

Model 1: adjusted for age and sex. Model 2: adjusted for age, sex, race and education. Model 3: adjusted for age, sex, race, education, smoke, BMI, CVD, hypertension and diabetes

*Abbreviations*: *CONUT* controlling nutritional status score, *COPD* chronic obstructive pulmonary disease, *HR* hazard ratio, *CI* confidence interval, *BMI* body mass index, *CVD* cardiovascular disease

### ROC analysis outcomes

**Fig. 2:**
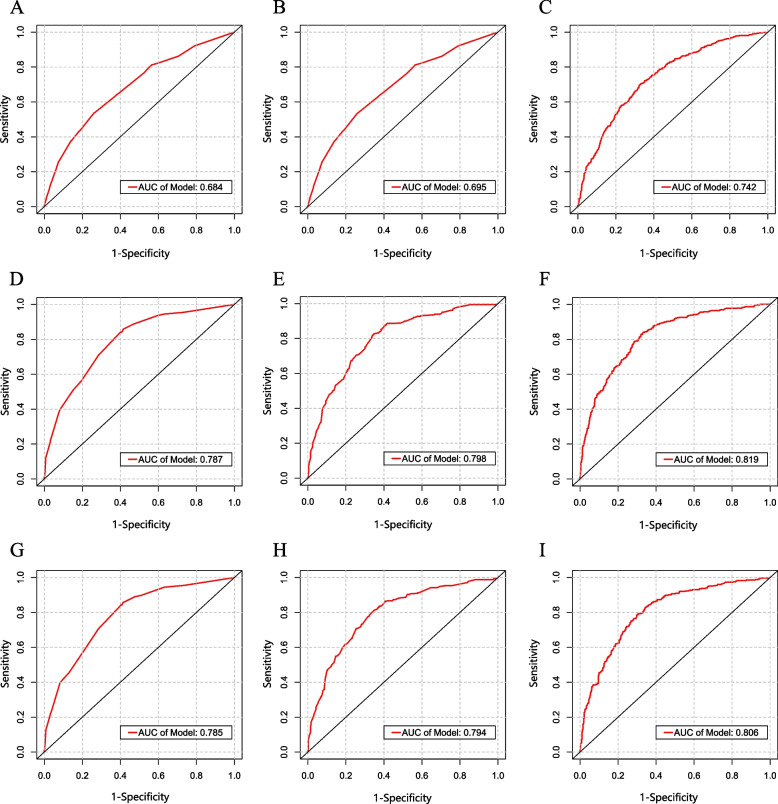
ROC curves of the CONUT score for predicting outcomes in COPD. **A**, **B** Prediction of CVD prevalence, adjusting the variables of Models 1-3 in Table 2. **D**-**I** Parts **D**-**F** and **G**-**I** predicted 10-year overall mortality and 10-year cardiovascular mortality, respectively, adjusted for the variables of Models 1-3 in Table 3. AUC, area under the receiver operating characteristic curve; CONUT, controlling nutritional status score; COPD, chronic obstructive pulmonary disease; CVD, cardiovascular disease

We plotted ROC curves of CONUT score for overall mortality and CVD mortality using both logistic regression models as well as Cox regression models mentioned above (Fig. 2). Figure 2A-C plots displayed the ROC curves examining the predictive capability of the CONUT score in relation to CVD prevalence. Among others, model 3 exhibited the superior forecast capability (AUC: 0.742, 95%CI: 0.716-0.768). CONUT score’s ROC analyses for the prediction of 5-year overall mortality and cardiovascular mortality were presented in Fig. 2D-F and G-I plots, respectively. Excellent predictive ability was demonstrated in model 3 for overall mortality (AUC: 0.819, 95%CI: 0.790-0.848) and model 3 for cardiovascular mortality (AUC: 0.806, 95%CI: 0.776-0.836).

### Subgroup analyses

Subgroup analyses were conducted in order to investigate the correlation between CONUT score and cardiovascular disease prevalence, cardiovascular mortality, and overall mortality (Additional file 5: Table S3). A substantial interaction was seen between the CONUT score and sex, which was shown to have an impact on CVD mortality. Regarding all-cause death, there was also a noteworthy interaction between CONUT scores with BMI. Among the variable BMI, underweight patients had a more significant correlation between CONUT scores and all-cause mortality than obese patients.

## Discussion

Malnutrition is defined as a score greater than two, according to the CONUT score [8]. We found that the prevalence of malnutrition among COPD patients was 21.31%, which was lower than Deng et al.'s reported prevalence [3]. It is likely that this difference arose because all of the individuals were in a state of stability and had a less severe disease than patients in a hospital. The incidence of malnutrition escalates in correlation with the advancing age of individuals diagnosed with COPD. This research presents novel findings indicating that the CONUT score has the potential to be a clinical predictive tool in assessing the prevalence of cardiovascular illness, CVD mortality, and overall mortality in COPD. In particular, the population suffering from malnutrition exhibited a 50% higher risk of all-cause mortality and an 86% higher likelihood of CVD mortality. Survival analyses showed that the CONUT score had a well-differentiated role in overall and CVD mortality. A greater risk of mortality was associated with higher CONUT scores in those with COPD, according to the subgroup analysis. This correlation was robust among men and underweight or obese individuals. Clinicians must, therefore, be cognizant of the elevated probability of death associated with this population.

Our investigation found a positive correlation between higher CONUT scores and increased all-cause death. Previous cross-sectional investigations indicated a correlation between the geriatric nutritional risk index (GNRI) and overall death among COPD individuals [23]. Our findings align with previous research, indicating that elevated CONUT scores were indicative of a suboptimal nutrition condition and an increased susceptibility to overall death. This highlights the need to evaluate the nutritional status when determining the likelihood of mortality in persons with COPD. Albumin serves as a fundamental component of the CONUT score, which plays a critical part in nutrition evaluation. Low albumin was linked to elevated rates of in-hospital mortality among COPD individuals, according to Dawei et al. [24]. Regarding the CONUT score, lymphocytes are immune cells that contribute to the regulation of inflammation. In elderly individuals with severe COPD, low lymphocyte counts were shown to correlate with a heightened risk of death, according to a prospective three-year study [25]. Zinellu et al. observed that the neutrophil-to-lymphocyte ratio (NLR) and the platelet-to-lymphocyte ratio (PLR) were shown to be significant predictors of unfavorable results in acute exacerbations of COPD [26]. This implies that individuals with COPD may have a heightened risk of mortality in relation to lower lymphocyte counts. The Cox regression analysis demonstrated a significant association between increased CONUT scores, which indicated diminished levels of albumin and lymphocyte counts, and a raised rate of overall death in COPD. It appeared that the CONUT score provided a more comprehensive prediction of COPD prognosis due to its combination of nutritional, immune, and inflammatory markers. Regular evaluation of the CONUT score has promise in assisting medical professionals in formulating suitable therapeutic interventions for individuals diagnosed with COPD, thereby reducing mortality.

A noteworthy association was identified between the CONUT score and CVD prevalence as well as CVD mortality in COPD individuals. COPD patients face a significantly elevated susceptibility to CVD, which is the most common complication of the disease [7]. The potential mechanisms linking CONUT scores and CVD events are intricate and diverse. There is, however, an association between each constituent of the CONUT score and cardiovascular disease. Elevated levels of cholesterol are well recognized as a significant risk variable contributing to the development and progression of CVD [27, 28]. Nevertheless, several studies have demonstrated that individuals suffering from chronic heart failure may experience impaired survival if their total cholesterol levels are low [29]. It can also be seen in patients with renal failure undergoing dialysis and in patients with malignant tumors [30, 31]. It seems that this lipid paradox may result from disturbed lipid metabolism caused by inflammation or malnutrition in the body, thereby increasing the risk of adverse effects [32]. As immune cells, lymphocytes are vital to the systemic inflammation response, and low lymphocyte counts contribute to the progression of atherosclerosis [33]. A notable association can be observed between increased NLR and PLR and adverse prognosis results in youthful individuals with acute myocardial infarction [34]. Thus, reduced lymphoid counts could be potentially linked to an elevated risk of CVD mortality. Lastly, chronic inflammation and malnutrition have an impact on the synthesis of albumin, which serves as a robust indicator of the onset of CVD in various patient populations. In addition, it is a highly accurate predictor of mortality from all causes and CVD [35]. Consequently, by combining these three metrics, the CONUT score may serve as a dependable biomarker for the identification of COPD patients who are at an elevated risk of CVD, as well as for the prediction of the mortality risk associated with the condition. The clinical management of patients can be guided by an initial evaluation of their nutritional, immunological, inflammatory, and lipid metabolic state, which can be accomplished through the use of the CONUT score.

As the name implies, the double burden of malnutrition occurs when malnutrition and obesity are simultaneously present, along with the associated non-communicable illnesses [36]. According to a current systematic review, the Asia-Pacific region bears an alarmingly heavy double burden of malnutrition, with obesity bearing a disproportionate share [37]. Chien et al. conducted a study that revealed a higher prevalence of CVD in obese malnourished adults exhibiting an elevated BMI and diminished serum albumin (ALB) levels compared to lean malnourished populations displaying lower BMI and ALB levels [38]. The study focused on asymptomatic Taiwanese adults. Our present investigation failed to identify a statistically significant interaction between BMI and CONUT score in relation to cardiovascular disease prevalence. However, when it came to all-cause mortality, there was a considerable interaction between the CONUT score and BMI (especially among patients who were wasting or obese). Consequently, additional investigation is necessary to ascertain whether the COPD population bears a double burden of malnutrition. It has been shown that nutritional supplementation improves a variety of COPD patient indicators, including respiratory muscle strength, exercise tolerance, quality of life, and the 6-min walk distance [5, 39]. A randomized clinical trial indicated that nutritional supplement therapy decreased 90-day mortality in hospitalized COPD or CVD patients [40]. Nutritional support is therefore regarded as an efficacious treatment for COPD. However, the impact of nutrient supplements taken orally on the progression of cardiovascular disease in individuals with COPD is uncertain. More exploration is required to ascertain the potential correlation between oral nutrient supplements and the formation and progression of CVD among COPD patients in order to establish whether such supplements provide positive effects.

There are several constraints within the scope of this research. First, the results apply primarily to the United States. Second, this study was a retrospective study, and certain variables were collected by validated questionnaires, leading to the potential for recall bias. Third, the impact of time variations in COUNT scores on all-cause and CVD mortality remains uncertain. Fourthly, we were unable to examine the association between varying COPD severities and the predictive power of the CONUT score for CVD prevalence and mortality. However, through a substantial sample derived from a nationwide cohort, our study demonstrates for the first time that the CONUT score might serve as a valuable predictive tool in assessing the CVD prevalence, overall death, and CVD mortality among persons with COPD. It provides an essential clinical management reference for early assessment of nutritional, immunologic, and validation profiles in individuals with COPD.

## Conclusions

In summary, the CONUT score provides an uncomplicated and readily attainable marker for forecasting CVD prevalence, total mortality, and mortality from CVD, in people who have received a diagnosis of COPD. The early evaluation of the CONUT score for the purpose of identifying COPD individuals at elevated risk could potentially be crucial in mitigating unfavorable clinical outcomes.

### Supplementary Information

Additional file 1: Figure S1: Bar graph illustrating prevalence of malnutrition (CONUT ≥ 2) by age group. CONUT, the controlling nutritional status score.

Additional file 2: Table S1: Univariate logistic analyses of the CONUT score and CVD prevalence in COPD.

Additional file 3: Table S2: Univariate COX analyses of the CONUT score and overall and cardiovascular mortality in COPD.

Additional file 4: Figure S2: Survival curves of the CONUT score risk classification system for all-cause mortality (A) and cardiovascular mortality (B) in COPD. CONUT, the controlling nutritional status score; COPD, chronic obstructive pulmonary disease.

Additional file 5: Table S3: Subgroup analyses of the relationship between the CONUT score and CVD prevalence, overall mortality and CVD mortality in COPD.

## Data Availability

No datasets were generated or analysed during the current study.

## Abbreviations

COPD: Chronic obstructive pulmonary disease
CVD: Cardiovascular disease
CONUT: Controlling nutritional status score
NHANES: National Health and Nutrition Examination Survey
ROC: Receiver operating characteristic curve
AUC: Area under the receiver operating characteristic curve
BMI: Body mass index
GNRI: Geriatric nutritional risk index
NLR: Neutrophil-to-lymphocyte ratio
PLR: Platelet-to-lymphocyte ratio
ALB: Serum albumin
SE: Standard error
OR: Odds ratio
HR: Hazard ratio
CI: Confidence interval
