# Supplementary figures and images for: Controlling nutritional status score in the prediction of cardiovascular disease prevalence, all-cause and cardiovascular mortality in chronic obstructive pulmonary disease population: NHANES 1999–2018
Source: BMC Pulm Med. 2024 Jul 24;24:356. doi: 10.1186/s12890-024-03175-7 (PMC11267957; doi:10.1186/s12890-024-03175-7)

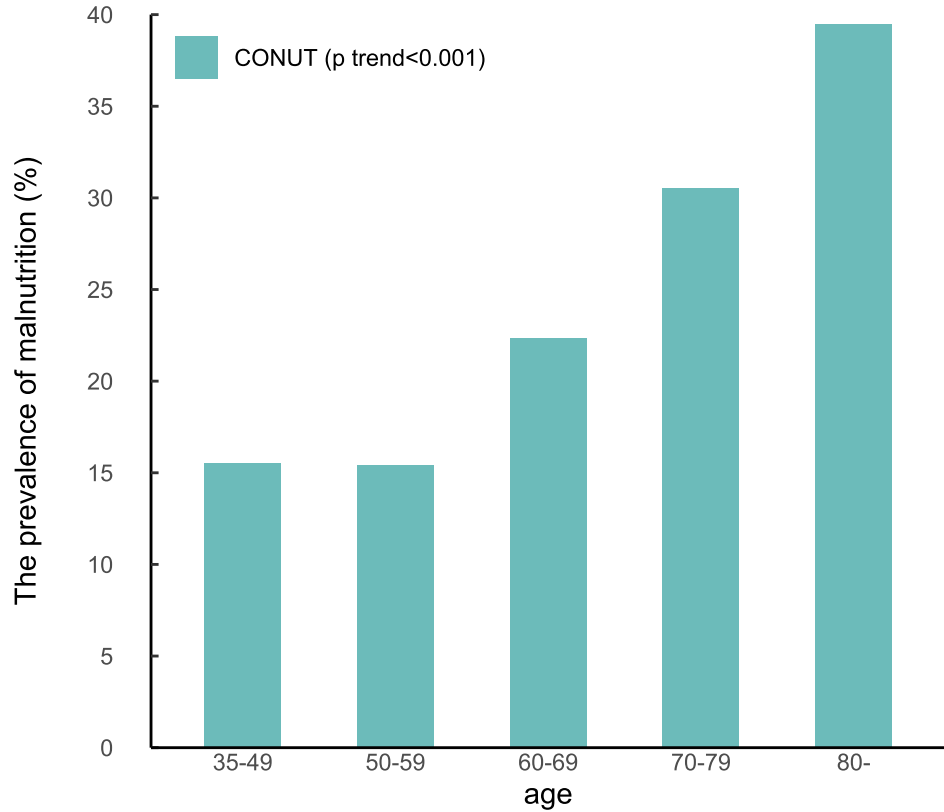

Supplement: Supplementary file 1 — Additional file 1: Figure S1: Bar graph illustrating prevalence of malnutrition (CONUT ≥ 2) by age group. CONUT, the controlling nutritional status score. [file 12890_2024_3175_MOESM1_ESM.pdf]

**A**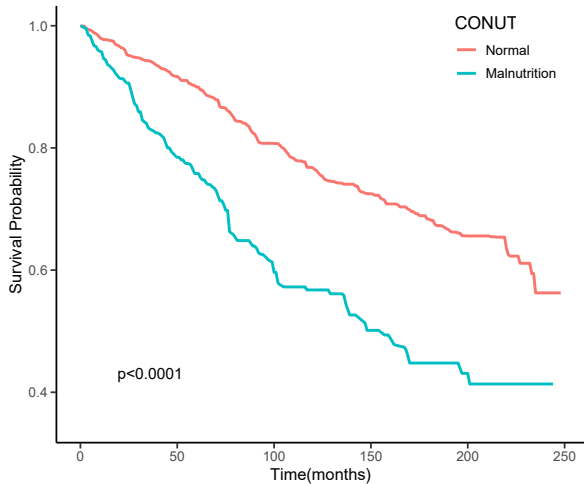**B**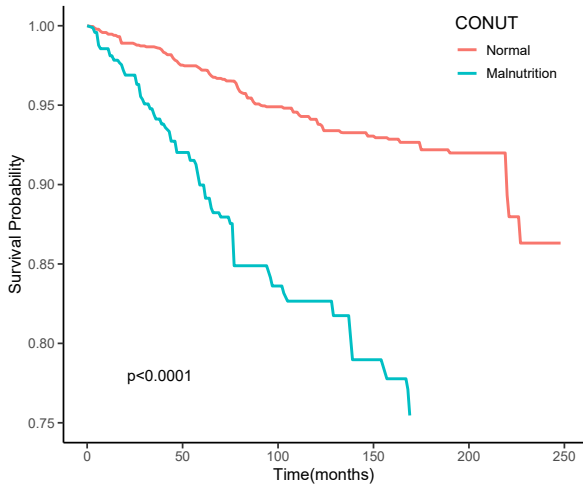

Supplement: Supplementary file 4 — Additional file 4: Figure S2: Survival curves of the CONUT score risk classification system for all-cause mortality (A) and cardiovascular mortality (B) in COPD. CONUT, the controlling nutritional status score; COPD, chronic obstructive pulmonary disease. [file 12890_2024_3175_MOESM4_ESM.pdf]
