# Supplementary material for: Controlling nutritional status score in the prediction of cardiovascular disease prevalence, all-cause and cardiovascular mortality in chronic obstructive pulmonary disease population: NHANES 1999–2018
Source: BMC Pulm Med. 2024 Jul 24;24:356. doi: 10.1186/s12890-024-03175-7 (PMC11267957; doi:10.1186/s12890-024-03175-7)
Supplement: Supplementary file 2 — Additional file 2: Table S1: Univariate logistic analyses of the CONUT score and CVD prevalence in COPD. [file 12890_2024_3175_MOESM2_ESM.docx]

**Table S1.** Univariate logistic analyses of the CONUT score and CVD prevalence in COPD.

| Characteristics | OR (95% CI) | *p* value |
| --- | --- | --- |
| CONUT score |  |  |
| <2 score | ref |  |
| ≥2 score | 2.37 (1.72-3.28) | <0.001 |
| Age |  |  |
| <60 | ref |  |
| ≥60 | 2.58 (1.94-3.44) | <0.001 |
| Sex |  |  |
| Male | ref |  |
| Female | 0.48 (0.36-0.63) | <0.001 |
| Race |  |  |
| Non-Hispanic white | ref |  |
| Non-Hispanic black | 1.15 (0.82-1.61) | 0.408 |
| Mexican American | 0.78 (0.45-1.33) | 0.352 |
| Other races | 1.04 (0.66-1.62) | 0.871 |
| BMI |  |  |
| Normal | ref |  |
| Underweight | 0.44 (0.14-1.36) | 0.153 |
| Overweight | 0.61 (0.40-0.92) | 0.019 |
| Obese | 0.83 (0.59-1.18) | 0.295 |
| Education |  |  |
| College graduate or above | ref |  |
| High school graduate or equivalent | 0.99 (0.67-1.47) | 0.962 |
| 9-11th grade | 1.36 (0.90-2.04) | 0.143 |
| Less than 9th grade | 2.32 (1.53-3.54) | <0.001 |
| Smoke |  |  |
| Never | ref |  |
| Former | 1.46 (1.02-2.10) | 0.040 |
| Current | 1.39 (0.98-1.96) | 0.063 |
| Hypertension |  |  |
| No | ref |  |
| Yes | 3.07 (2.14-4.41) | <0.001 |
| Diabetes |  |  |
| No | ref |  |
| Yes | 2.52 (1.80-3.53) | <0.001 |
| Asthma |  |  |
| No | ref |  |
| Yes | 1.34 (0.99-1.82) | 0.059 |

Abbreviations: CONUT, controlling nutritional status score; CVD, cardiovascular disease; COPD, chronic obstructive pulmonary disease; OR, odds ratios; CI, confidence interval; BMI, body mass index.
