# Supplementary material for: Controlling nutritional status score in the prediction of cardiovascular disease prevalence, all-cause and cardiovascular mortality in chronic obstructive pulmonary disease population: NHANES 1999–2018
Source: BMC Pulm Med. 2024 Jul 24;24:356. doi: 10.1186/s12890-024-03175-7 (PMC11267957; doi:10.1186/s12890-024-03175-7)
Supplement: Supplementary file 3 — Additional file 3: Table S2: Univariate COX analyses of the CONUT score and overall and cardiovascular mortality in COPD. [file 12890_2024_3175_MOESM3_ESM.docx]

**Table S2.** Univariate COX analyses of the CONUT score and overall and cardiovascular mortality in COPD.

| Characteristics | All-cause mortality | |  | Cardiovascular mortality | |
| --- | --- | --- | --- | --- | --- |
|  | HR (95% CI) | *p* value |  | HR (95% CI) | *p* value |
| CONUT score |  |  |  |  |  |
| <2 score | ref |  |  | ref |  |
| ≥2 score | 2.18 (1.67-2.84) | <0.001 |  | 3.36 (2.21-5.12) | <0.001 |
| Age |  |  |  |  |  |
| <60 | ref |  |  | ref |  |
| ≥60 | 5.33 (3.94-7.21) | <0.001 |  | 12.86 (5.74-28.83) | <0.001 |
| Sex |  |  |  |  |  |
| Male | ref |  |  | ref |  |
| Female | 0.48 (0.38-0.61) | <0.001 |  | 0.41 (0.27-0.63) | <0.001 |
| Race |  |  |  |  |  |
| Non-Hispanic white | ref |  |  | ref |  |
| Non-Hispanic black | 0.72 (0.52-1.00) | 0.050 |  | 0.66 (0.38-1.15) | 0.144 |
| Mexican American | 0.46 (0.27-0.80) | 0.006 |  | 0.45 (0.18-1.11) | 0.083 |
| Other races | 0.44 (0.27-0.72) | 0.001 |  | 0.46 (0.23-0.93) | 0.029 |
| BMI |  |  |  |  |  |
| Normal | ref |  |  | ref |  |
| Underweight | 1.08 (0.57-2.06) | 0.808 |  | 0.50 (0.16-1.59) | 0.240 |
| Overweight | 0.62 (0.45-0.84) | 0.003 |  | 0.57 (0.36-0.91) | 0.019 |
| Obese | 0.66 (0.49-0.89) | 0.006 |  | 0.75 (0.47-1.21) | 0.245 |
| Education |  |  |  |  |  |
| College graduate or above | ref |  |  | ref |  |
| High school graduate or equivalent | 1.31 (0.98-1.75) | 0.066 |  | 1.38 (0.73-2.60) | 0.317 |
| 9-11th grade | 1.33 (0.96-1.85) | 0.087 |  | 2.01 (1.08-3.74) | 0.027 |
| Less than 9th grade | 2.16 (1.55-2.99) | <0.001 |  | 4.56 (2.56-8.11) | <0.001 |
| Smoke |  |  |  |  |  |
| Never | ref |  |  | ref |  |
| Former | 2.31 (1.73-3.10) | <0.001 |  | 1.77 (1.07-2.94) | 0.027 |
| Current | 1.57 (1.12-2.20) | 0.008 |  | 0.98 (0.54-1.79) | 0.943 |
| Cardiovascular disease |  |  |  |  |  |
| No | ref |  |  | ref |  |
| Yes | 2.79 (2.17-3.57) | <0.001 |  | 5.57 (3.88-8.00) | <0.001 |
| Hypertension |  |  |  |  |  |
| No | ref |  |  | ref |  |
| Yes | 1.54 (1.18-2.02) | 0.002 |  | 2.61 (1.68-4.05) | <0.001 |
| Diabetes |  |  |  |  |  |
| No | ref |  |  | ref |  |
| Yes | 1.84 (1.42-2.39) | <0.001 |  | 2.41 (1.46-3.99) | <0.001 |
| Asthma |  |  |  |  |  |
| No | ref |  |  | ref |  |
| Yes | 0.88 (0.69-1.13) | 0.319 |  | 0.84 (0.53-1.33) | 0.457 |

Abbreviations: CONUT, controlling nutritional status score; COPD, chronic obstructive pulmonary disease; HR, hazard ratio; CI, confidence interval; BMI, body mass index.
