# Supplementary material for: Controlling nutritional status score in the prediction of cardiovascular disease prevalence, all-cause and cardiovascular mortality in chronic obstructive pulmonary disease population: NHANES 1999–2018
Source: BMC Pulm Med. 2024 Jul 24;24:356. doi: 10.1186/s12890-024-03175-7 (PMC11267957; doi:10.1186/s12890-024-03175-7)
Supplement: Supplementary file 5 — Additional file 5: Table S3: Subgroup analyses of the relationship between the CONUT score and CVD prevalence, overall mortality and CVD mortality in COPD. [file 12890_2024_3175_MOESM5_ESM.docx]

**Table S3.** Subgroup analyses of the relationship between the CONUT score and CVD prevalence, overall mortality and CVD mortality in COPD.

| Subgroups | Number (%) | Prevalence of CVD | | |  | All-cause mortality | | |  | Cardiovascular mortality | | |
| --- | --- | --- | --- | --- | --- | --- | --- | --- | --- | --- | --- | --- |
|  |  | OR (95% CI) | *p* value | *p* for interaction |  | HR (95% CI) | *p* value | *p* for interaction |  | HR (95% CI) | *p* value | *p* for interaction |
| Age |  |  |  | 0.393 |  |  |  | 0.381 |  |  |  | 0.719 |
| <60 | 657 (53.27) | 1.71 (0.91-3.19) | 0.094 |  |  | 2.11 (1.13-3.94) | 0.019 |  |  | 2.87 (0.59-14.00) | 0.192 |  |
| ≥60 | 844 (46.73) | 2.34 (1.57-3.50) | <0.001 |  |  | 1.62 (1.25-2.09) | <0.001 |  |  | 2.43 (1.64-3.60) | <0.001 |  |
| Sex |  |  |  | 0.306 |  |  |  | 0.054 |  |  |  | 0.015 |
| Male | 632 (37.72) | 2.85 (1.69-4.80) | <0.001 |  |  | 2.75 (1.98-3.81) | <0.001 |  |  | 5.07 (2.90-8.85) | <0.001 |  |
| Female | 869 (62.28) | 1.87 (1.11-3.16) | 0.019 |  |  | 1.59 (1.04-2.44) | 0.031 |  |  | 1.73 (0.84-3.55) | 0.135 |  |
| Race |  |  |  | 0.144 |  |  |  | 0.157 |  |  |  | 0.200 |
| Non-Hispanic white | 933 (79.44) | 2.44 (1.65-3.61) | <0.001 |  |  | 2.07 (1.51-2.83) | <0.001 |  |  | 3.64 (2.27-5.84) | <0.001 |  |
| Non-Hispanic black | 243 (8.36) | 1.06 (0.53-2.12) | 0.873 |  |  | 2.83 (1.38-5.81) | 0.005 |  |  | 1.50 (0.42-5.45) | 0.534 |  |
| Mexican American | 131 (3.02) | 2.60 (0.70-9.74) | 0.141 |  |  | 2.42 (0.94-6.24) | 0.067 |  |  | 0.00 (0.00-0.00) | <0.001 |  |
| Other races | 194 (9.18) | 3.60 (1.57-8.28) | 0.004 |  |  | 6.07 (2.11-17.43) | <0.001 |  |  | 6.73 (1.23-36.73) | 0.028 |  |
| BMI |  |  |  | 0.149 |  |  |  | 0.014 |  |  |  | 0.158 |
| Normal | 306 (20.72) | 2.58 (1.23-5.40) | 0.013 |  |  | 1.53 (0.88-2.67) | 0.134 |  |  | 2.00 (0.81-4.95) | 0.131 |  |
| Underweight | 44 (3.51) | 11.44 (0.44-296.86) | 0.084 |  |  | 4.88 (1.72-13.86) | 0.003 |  |  | 5.11 (0.80-32.57) | 0.084 |  |
| Overweight | 438 (29.72) | 1.50 (0.87-2.56) | 0.140 |  |  | 1.25 (0.74-2.12) | 0.401 |  |  | 2.36 (1.14-4.89) | 0.021 |  |
| Obese | 713 (46.05) | 2.70 (1.52-4.80) | <0.001 |  |  | 3.02 (2.08-4.40) | <0.001 |  |  | 4.87 (2.57-9.23) | <0.001 |  |
| Education |  |  |  | 0.223 |  |  |  | 0.335 |  |  |  | 0.158 |
| College graduate or above | 653 (47.69) | 2.10 (1.29-3.41) | 0.003 |  |  | 2.20 (1.46-3.30) | <0.001 |  |  | 5.62 (2.54-12.48) | <0.001 |  |
| High school graduate or equivalent | 380 (28.54) | 1.73 (0.98-3.06) | 0.059 |  |  | 2.23 (1.31-3.78) | 0.003 |  |  | 2.40 (0.85-6.76) | 0.099 |  |
| 9-11th grade | 276 (16.46) | 3.81 (1.86-7.78) | <0.001 |  |  | 1.56 (0.83-2.92) | 0.168 |  |  | 1.66 (0.69-4.03) | 0.259 |  |
| Less than 9th grade | 192 (7.30) | 3.88 (1.36-11.05) | 0.013 |  |  | 3.68 (2.18-6.21) | <0.001 |  |  | 3.99 (1.94-8.24) | <0.001 |  |
| Smoke |  |  |  | 0.332 |  |  |  | 0.512 |  |  |  | 0.371 |
| Never | 441 (28.23) | 1.64 (0.85-3.16) | 0.137 |  |  | 1.70 (0.87-3.32) | 0.120 |  |  | 2.26 (0.82-6.20) | 0.113 |  |
| Former | 555 (35.03) | 2.74 (1.72-4.36) | <0.001 |  |  | 2.25 (1.52-3.34) | <0.001 |  |  | 3.20 (1.65-6.19) | <0.001 |  |
| Current | 505 (36.74) | 3.10 (1.55-6.20) | 0.002 |  |  | 2.73 (1.62-4.61) | <0.001 |  |  | 5.17 (2.00-13.38) | <0.001 |  |
| Hypertension |  |  |  | 0.529 |  |  |  | 0.080 |  |  |  | 0.110 |
| No | 594 (45.34) | 2.61 (1.43-4.78) | 0.002 |  |  | 1.48 (0.88-2.48) | 0.140 |  |  | 1.72 (0.69-4.29) | 0.244 |  |
| Yes | 907 (54.66) | 2.04 (1.31-3.17) | 0.002 |  |  | 2.57 (1.90-3.48) | <0.001 |  |  | 3.99 (2.50-6.38) | <0.001 |  |
| Diabetes |  |  |  | 0.391 |  |  |  | 0.200 |  |  |  | 0.406 |
| No | 1165 (82.00) | 2.06 (1.38-3.09) | <0.001 |  |  | 1.94 (1.44-2.60) | <0.001 |  |  | 2.86 (1.68-4.86) | <0.001 |  |
| Yes | 336 (18.00) | 2.83 (1.61-5.00) | <0.001 |  |  | 2.86 (1.86-4.39) | <0.001 |  |  | 4.21 (1.92-9.23) | <0.001 |  |
| Asthma |  |  |  | 0.388 |  |  |  | 0.267 |  |  |  | 0.475 |
| No | 860 (57.38) | 2.71 (1.75-4.20) | <0.001 |  |  | 2.49 (1.75-3.53) | <0.001 |  |  | 3.90 (2.21-6.89) | <0.001 |  |
| Yes | 641 (42.62) | 2.00 (1.20-3.34) | 0.009 |  |  | 1.82 (1.15-2.88) | 0.010 |  |  | 2.67 (1.27-5.61) | 0.009 |  |

Abbreviations: CONUT, controlling nutritional status score; COPD, chronic obstructive pulmonary disease; CVD, cardiovascular disease; OR, odds ratios; HR, hazard ratio; CI, confidence interval; BMI, body mass index.
